# Early patello-femoral condropathy assessment through quantitative analyses via T2 mapping magnetic resonance after anterior cruciate ligament reconstruction

**DOI:** 10.1007/s11547-023-01716-4

**Published:** 2023-10-03

**Authors:** Domenico Zagaria, Pietro Costantini, Ilaria Percivale, Flavia Abruzzese, Gloria Ghilardi, Marco Landrino, Mauro Porta, Massimiliamo Leigheb, Alessandro Carriero

**Affiliations:** 1 Department of Radiology, Università Degli Studi del Piemonte Orientale, Alessandria, Italy; 2 Department of Orthopedics and Rehabilitation, Università Degli Studi del Piemonte Orientale, Alessandria, Italy; 3 Department of Orthopedics and Rehabilitation, Presidio Ospedaliero SS. Trinità Di Borgomanero, ASL Novara, Borgomanero, Italy

**Keywords:** Magnetic resonance, T2 mapping, Patello-femoral chondropathy, Osteoarthrosis, Anterior cruciate ligament, Reconstruction

## Abstract

**Background:**

Patellar femoral chondropathy (FPC) is a common problem in patients undergoing anterior cruciate ligament reconstruction (ACL-R) surgery, which, if left untreated, predisposes to arthrosis. Magnetic resonance imaging (MRI) is the non-invasive gold standard for morphological evaluation of cartilage, while in recent years advanced MRI techniques (such as T2 mapping) have been developed to detect early cartilage biochemical changes. This study evaluates the different onset of early PFC between B-TP-B and HT through T2 mapping. Secondly, it aims to assess the presence of any concordance between self-reported questionnaires and qualitative MRI.

**Materials and methods:**

19 patients enrolled were divided into two groups based on the type of intervention: B-PT-B and HT. After a median time of 54 months from surgery, patients were subjected to conventional MRI, T2 mapping, and clinical-functional evaluation through three self-reported questionnaires: Knee Injury and Osteoarthritis index (KOOS); Tegner Lysholm Knee Scoring Scale; International Knee Documentation Committee (IKDC).

**Results:**

There is not statistically significant difference in the comparison between the two MRI techniques and the two reconstructive techniques. KOOS and Tegner Lysholm scales showed significant agreement with MRI results on the grading of chondropathy.

**Conclusions:**

There are no differences between B-TP-B and HT techniques in the early development of PFC detectable through non-invasive methods. Due to the large reduction in the frequency of physical activity following ACL-R and the finding of mild PFC (grade I and II) in a substantial proportion of patients, after a relatively short period from ACL-R, all patients should undergo conservative treatment.

## Introduction

Osteoarthritis is a well-renowned chronic pathology that involves joints and tissues. Beginning with progressive damaging of the articular cartilage and evolving to the subchondral bone and surrounding synovial structures, it is one of the most common causes of disability worldwide [1].

In knee surgery, Anterior Cruciate Ligament Reconstruction (ACL-R) is usually linked to long term onset of arthrosis. Giorgino et al., reported that after ACL rupture there is a fourfold increase in risk of developing osteoarthritis [2].

Reconstruction is done either by bone-patellar tendon-bone (B-PT-B) or with two hamstring tendons (HT): semitendinosus and gracilis.

B-TP-B has been popularized in the ‘60 s by Erikson [3] and offers a fast procedure that can lead to a reprise of the knee functionality as before of the trauma and a reduced fracture risk. On the contrary, B-TP-B reduces the anterior knee pain, at the cost of a reduction of the extensor apparatus power [4–9].

Studies demonstrated that patello-femoral arthrosis (PFA) in ACL-R patients is almost inevitable as it has been reported in up to 80% of the patients after 10 years and up to 90% after 15 years from the surgery [10, 11].

PFA is most common in elderly, but can be found also in athletes [12, 13]. This condition is often underdiagnosed as follow-ups are usually performed by radiography in which only advanced arthrosis is recognisable. Plain radiography do not show statistically significant differences between HT and B-PT-B.

recostruction [14]. As detecting early chondropathy in a reversible phase is crucial for personalized interventions, radiography is not ideal [15–20].

Magnetic Resonance (MR) is the non-invasive gold standard imaging technique for chondral anatomy and the evaluation of early bone insults (eg presence of oedema at the spongy bone). However, MR has less sensitivity in picturing chondral biochemical alterations in early chondropathy stage, such as reduction of proteoglycans and changes in water and collagen percentage [21–24]. Different studies on cartilage detriment following ACL reconstruction report how in 1–5 years follow ups patellofemoral cartilage (PFC) is compromised way more than tibiofemoral cartilage [25, 26].

Lately, different MR sequences such as T1r, T2 and dGEMRIC has been developed for evaluating changes in cartilage composition before morphologic changes can be appreciated by standard MR imaging (MRI) [27]. In particular, studies linking variations in T2 values and variations of water and collagen in cartilage have shown significant differences between symptomatic and asymptomatic patients with arthrosis [28, 29].

T2 mapping has been validated for evaluating early degenerative changes in various joints such as knee, wrist and ankle [28, 30, 31]. During T2 mapping acquisition, the T2 relaxation time of the cartilage is measured by using fixed repetition time and multiple spin-echo times. T2 mapping values tend to increase with the aging of the patient or with disruptive processes [29]. Changes in T2 mapping values do not correlate with specific pathologies but underlines biochemical alterations within the cartilage.

This study firstly aims to assess the possible presence of differences in the onset of chondropathy between the use of B-TP-B and HT for ACL-R comparing T2 mapping evaluation and standard MR morphologic evaluation. Secondarily, it aims to understand whether there are specific zones in the PFC more prone to detriment. We choose to divide the PFC in 9 compartments following the 3-dimensional axes: superior—middle—inferior, lateral—central—medial; superficial—intermediate—deep.

## Materials and methods

This prospective study has been conducted in accordance with the principles of the Declaration of Helsinki. Informed consent has been signed by patients enrolled in the study. We enrolled 19 patients sorted in two groups: patients who underwent an ACL reconstruction with B-TP-B (group 1; 9 patients) and with HT (group 2, 10 patients) between June 2016 and January 2020. All patients underwent surgery at the same Orthopaedic Center (Ospedale Maggiore della Carità—Novara—Italy). We used the following inclusion criteria: patients between 18- and 40-year-old, ACL rupture without other ligament lesions, surgery done within 6 months from trauma, surgery done in the last 6 years, no other knee traumas since the surgery. The 6-year-from-surgery cut off represents an optimal timeframe for the interception of early chondropathy [7]. The exclusion criteria were: Body Mass Index (BMI) > 30 kg/m2, presence of any kind of lesions at the cartilage at the moment of surgery, disease at the PF joint, genu valgus, genu varus, instability of the operated knee and refuse to participate. The presence of damages at the cartilage (e.g. chondral fissuring) was visualised via qualitative assessment through pre-operation MRI. Patients > 40 years were excluded to reduce the influence of age-related osteoarthritis [32].

All tests were performed on a 1.5T (Philips Ingenia Ambition) with a 16-channel coil dedicated for the knee. The sequences performed were T1-weighted on the sagittal plane (FOV 140 × 140 mm, TR/TE/NEX 525/min full/2, matrix 320 × 224, slice thickness 4 mm, gap 0.4 mm), T2-weighted on the axial plane (FOV 140 × 140 mm, TR/TE/NEX 6800/102/2, matrix 320 × 224, slice thickness 4 mm, 0.4 mm), T2 FAT-SAT on the coronal plane (FOV 140 × 140 mm, TR/TE/NEX 6800/102/2, matrix 320 × 224, slice thickness 4 mm, gap 0.4 mm) and proton density (PD) FAT-SAT on the sagittal plane (FOV 140 × 140 mm, TR/TE/NEX 1640/35/2, matrix 320 × 224, slice thickness 4 mm, gap 0.4 mm). T2 mapping was performed via multi-echo spin-echo (SE) sequence called T2-Cal (FOV 140 × 140 mm, TR/TE 1000/7.6, 15.2, 22.8, 30.4, 37.6, 45.6, 52.8 e 60.6 ms, matrix 320 × 224, slice thickness 3 mm, gap 0 mm, bandwidth 228 kHz).

Every patient enrolled has been visited the day of the MR acquisition by our orthopaedic team who registered the most valuable anamnesis data (sex, age, operated knee, date of surgery, comorbidity), and administered three self-reported questionnaires: Knee Injury and Osteoarthritis index (KOOS) (score 0–100); Tegner Lysholm Knee Scoring Scale (score 0–100); International Knee Documentation Committee (IKDC) (score 0–100). Physical activity pre- and post-surgery was evaluated via Bhapkar test and non-parametric Wilcoxon test. Results from the group’s analysis via qualitative MRI and three functional scales (KOOS, Tegner Lysholm, IKDC) were compared via non-parametric Wilcoxon Rank Sum test.

### Image analysis

**Fig. 1:**
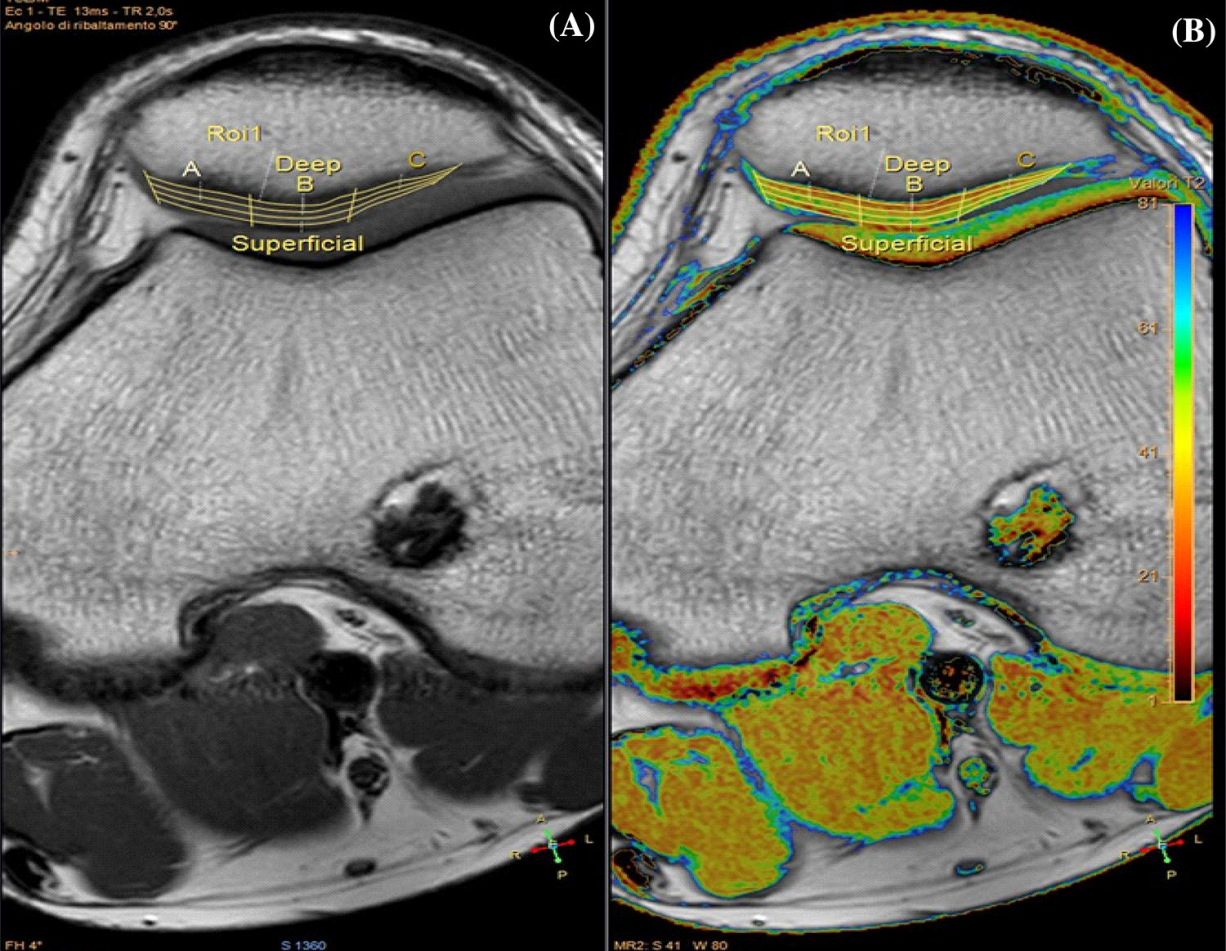
T2 mapping: **a** segmentation of the cartilage; **b** colorimetric map made by the software

**Fig. 2:**
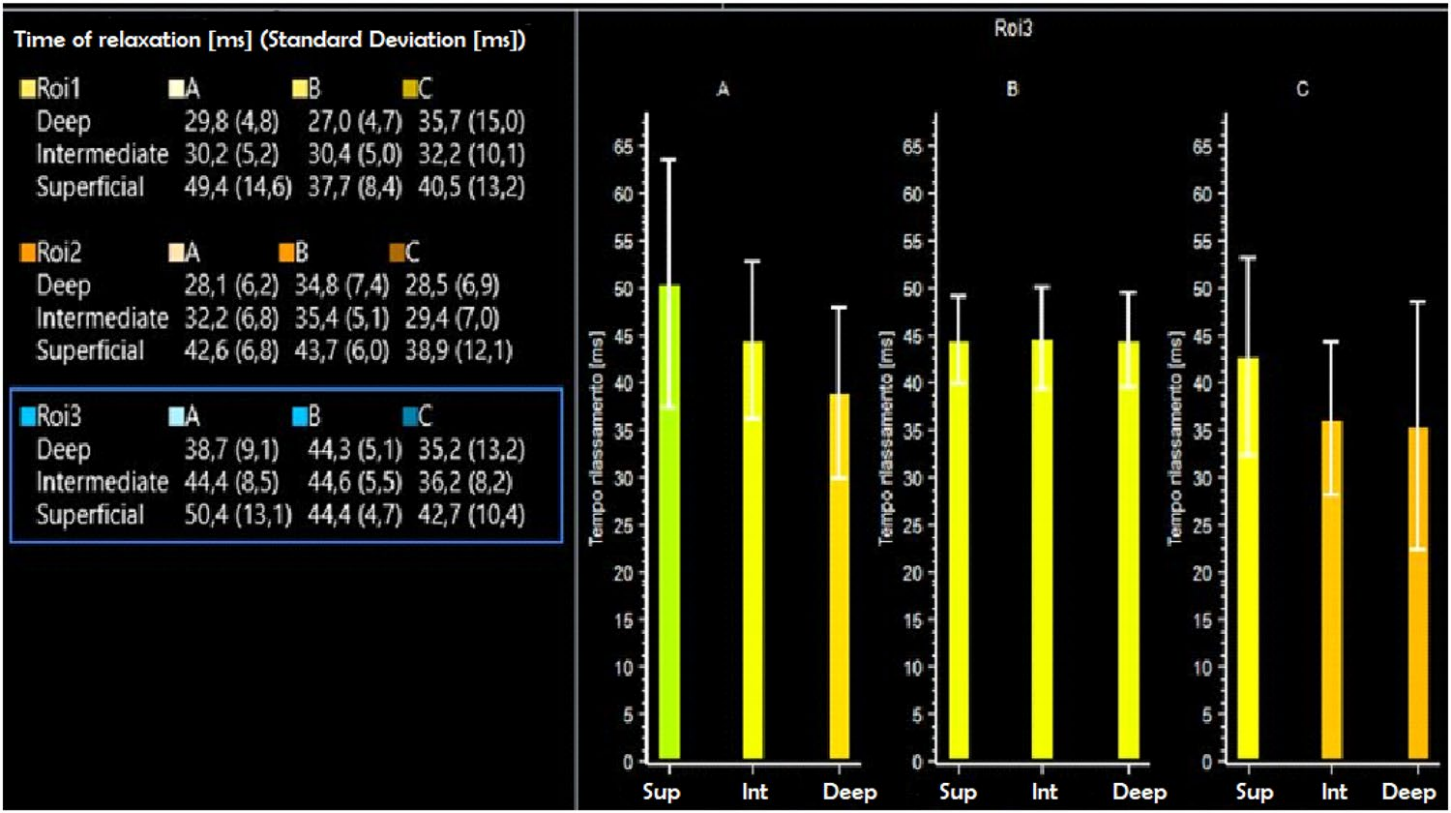
T2 mapping values of the 27 volumes in which the PF cartilage was divided. The trochlear part of the PF cartilage was excluded due to its small size thus leading to approximative segmentation. Roi1 = Superior; Roi2 = Medium; Roi3: Inferior. Sup = superior; Int = intermediate

Images were anonymized, randomized and read by two musculoskeletal experts (with 5 and 30 years of experience respectively). Images were evaluated through a morphological analysis of the cartilage following the modified Outerbridge classification [33] and a quantitative analysis: 11 patients presented grade 0-I chondropathy and 8 patients presented grade II chondropathy. The quantitative analysis was performed through T2 mapping sequence evaluation via Philips IntelliSpace Portal v.93. The comparison between the groups was done on 9 segments obtained by dividing the cartilage cranio-caudally, latero-laterally and antero-posteriorly and by considering 27 segments of the PF joint. Manual segmentation was performed in the presence of both musculoskeletal experts with intra-procedural agreement. Cartilage was selected by tracing two lines: one at the bone-cartilage interface and one at the cartilage-joint interface. Cartilage was divided cranio-caudally (superior, middle, inferior), latero-laterally (lateral, central, medial) and antero-posteriorly (superficial, intermediate, deep) thus obtaining 27 volumes (Fig. 1). The software produces a colorimetric map based on T2 relaxation time ranging from 0 ms (in blue) to 80 ms (in dark red) with a relative T2 mapping mean value for every segment (Fig. 2).

## Results

### Population

Patients were divided into two grups: group 1 (9 patients who underwent ACL reconstruction with B-TP-B) and group 2 (10 patients who underwent ACL reconstruction with HT). Group 1 comprehended 8 males and 1 female while group 2 comprehended 8 males and 2 females. Due to the small population, we did not compare the two groups by age, BMI, and years from surgery, but instead we considered the single group as a whole. Table 1 reports general information about the two groups. Group 1 (B-TP-B) had a mean age of 26.67 years, with the youngest patient being 21 and the oldest one being 33. The mean BMI was 25.37 kg/m^2^. The lower BMI was 19.50 kg/m^2^ while the highest was 29.10 kg/m^2^. Mean time from surgery was 51.996 months. The shortest period from surgery was 36 months, while the longest period was 72 months.

**Table 1:** B-TP-B: bone-patellar tendon-bone; HT: Hamstring Tendon

Group (n° pt)	Summary	Age (years)	BMI (kg/m^2^)	Time from surgery (months)
B-TP-B (9)	Mean ± SD	26.67 ± 4.472	25.37 ± 2.963	51.996 ± 10.392
	Min	21.00	19.50	36
	Max	33.00	29.10	72
HT (10)	Mean ± SD	29.00 ± 3.768	25.34 ± 2.194	56.4 ± 10.8
	Min	22.00	20.50	36
	Max	35.00	28.40	72
Total (19)	Mean ± SD	27.89 ± 4.267	25.36 ± 2.564	54.312 ± 10.86
	Min	21.00	19.50	36
	Max	35.00	29.10	72

Table 1 reports a concise summary of the two groups (B-TP-B and HT) and the population. Mean age, as well as minimum (min) and maximus (max) age are expressed in years. Time from surgery is expressed in months. n° pt = number of patients. SD = Standard deviation

Group 2 (HT) had a mean age of 29 years, ranging from 22 to 35 years. Mean BMI was 25.34 kg/m^2^, with the lower BMI being 20.50 kg/m^2^ and the higher 28.40 kg/m^2^. Mean time from surgery of the second group was 54.312 months. The shortest period from surgery was 36 months and the longest one was 72 months.

### Functional assessment

The physical activity pre- and post-surgery of the 19 patients was evaluated via Bhapkar test and non-parametric Wilcoxon test (Table 2). Nearly every patient reduced physical activity. Only 7 patients who used to perform physical activity more than twice per week did not change weekly physical activity. On the contrary, 1 patient who lived a sedentary life before the surgery increased physical activity post-surgery (twice per week). The reduction of the physical activity of the patients was statistically significant (p-value < 0.05).

**Table 2:** Here reported are the changes in physical activity for the 19 patients

Physical activity pre surgery	Physical activity post surgery			Total	Bhapkar Test	Wilcoxon Test
	0/ week	≤ 2/ week	> 2/ week			
0/week	1	0	1	2	.020	.011
≤ 2/week	3	0	0	3		
> 2/ week	4	3	7	14		
Total	8	3	8	19		

### Qualitative and quantitative MRI assessment

The non-parametric Wilcoxon Rank Sum test regarding the results obtained by qualitative and quantitative MRI between group 1 and group 2 showed no significant differences. In Table 3 we report the comparison between qualitative MRI and quantitative T2 mapping MRI in describing two independent groups that underwent ACL-R with B-TP-B (Group 1) and HT (Group 2). Here reported is the comparison between the 9 segments.

**Table 3:** Comparison between the two groups (B-TP-B vs HT) through conventional MRI assessment and T2 mapping assessment of the PF joint. Mean value of all the values reported

Wilcoxon Rank Sum test between independent groups:			
Group 1 (B-TP-B) vs Group 2 (HT)			
Variable	Group 1 (B-TP-B) (median)	Group 2 (HT) (median)	P-value
Superior	37.20	40.64	0.289
Middle	36.08	34.36	0.935
Inferior	37.53	35.62	0.807
Lateral	35.22	38.51	0.744
Central	36.12	38.11	0.683
Medial	35.58	37.32	0.624
Superficial	31.92	33.14	0.807
Intermediate	34.99	36.28	0.935
Deep	43.26	43.71	0.744
Mean value	36.63	38.44	0.744

Furthermore, we compared two independent groups: patients with grade 0/I chondropathy and patients with grade II chondropathy and their results from the three functional scales (KOOS, Tegner Lysholm, IKDC). KOOS and Tegner Lysholm scales were found concordant to qualitative MRI as reported in Table 4 with a p-value < 0.05.

**Table 4:** Comparison between KOOS, Tegner Lysholm and IKDC scales with qualitative MRI

Non-parametric Wilcoxon Rank Sum test between indipendent groups: grade 0-I chondropathy vs grade II chondropathy			
Variable	Grade		P-value
	0-I	II	
KOOS	89.00 (15)	80.00 (7)	0.047
Tegner Lysholm	95.00 (14)	80.50 (7.5)	0.014
IKDC	86.60 (13.8)	74.10 (13.25)	0.120

Quantitative MRI evaluation of the cartilage’s segments and functional scales were compared through Spearman’s Index showing no correlation (Table 5). Table 5 shows all the data relative to the 27 segments in which the PF joint was divided. No significant concordance was found between T2 mapping MRI evaluation of the single segment and the functional scales.

**Table 5:** KOOS: Knee Injury and Osteoarthitis index; IKDC: International Knee Documentation Committee; S = Superior; M = Middle; I = Inferior; A = Laterale; B = Central; C = Medial; Sup = Superficial; Int = Intermediate

Spearman Index (grouped data)			
Var1-Var2	Numero	rho	P-value
KOOS-S	19	0.031	0.900
KOOS-M	19	− 0.153	0.532
KOOS-I	19	− 0.041	0.870
KOOS-A	19	− 0.033	0.895
KOOS-B	19	0.002	0.994
KOOS-C	19	0.020	0.935
KOOS-sup	19	0.04	0.878
KOOS-int	19	0.001	0.980
KOOS-prof	19	− 0.120	0.626
KOOS-mean value	19	0.010	0.967
Tegner Lysholm-S	19	0.050	0.840
Tegner Lysholm-M	19	− 0.138	0.572
Tegner Lysholm-I	19	− 0.252	0.298
Tegner Lysholm-A	19	− 0.193	0.430
Tegner Lysholm-B	19	− 0.069	0.778
Tegner Lysholm-C	19	− 0.073	0.767
Tegner Lysholm-sup	19	− 0.044	0.857
Tegner Lysholm-int	19	− 0.107	0.664
Tegner Lysholm-prof	19	− 0.212	0.383
Tegner Lysholm-mean value	19	− 0.138	0.574
IKDC-S	19	− 0.081	0.742
IKDC-M	19	− 0.248	0.306
IKDC-I	19	− 0.196	0.421
IKDC-A	19	− 0.215	0.378
IKDC-B	19	− 0.166	0.496
IKDC-C	19	− 0.011	0.966
IKDC-sup	19	− 0.123	0.616
IKDC-int	19	− 0.172	0.480
IKDC-deep	19	− 0.244	0.315
IKDC-mean value	19	− 0.145	0.553

## Discussions

Following the rupture of the anterior cruciate ligament, its reconstruction represents the best therapeutic option especially in young patients. Unlikely, this intervention is burdened by a high rate of development of early osteoarthritis that leads to chronic pain, particularly in the patellofemoral compartment. Since its diagnosis is often delayed in routine follow-up instrumental examinations, our study aimed to use the advanced T2 mapping magnetic resonance technique to intercept early changes of the osteocartilaginous layer in this specific compartment. Zhao et al., demonstrated how MRI is capable of measuring early biochemical changes in the cartilage via T2 mapping [34]; his early detection can lead to anticipated and more personalized treatments leading to better outcomes for the patient by slowing chondropathy [2].

This study considered 19 patients (16 males and 3 females, with an average age of about 26.7 years), after an average time of about 54 months from surgery. After the intervention we found a statistically significant reduction in weekly physical activity. Patients with ACL reconstruction may not return to preinjury levels of sports participation for a variety of reasons. As Ardern et al. [35] reported, the most common cause of the reduction are: fear of reinjury (19%), reasons other than the knee (18%), knee impairments (13%), and changes in lifestyle (11%).

In our group of patients, we have found that the reduced functionality can be linked to the development of patello-femoral chondropathy. T2 mapping technique allows its detection in 57.9% of patients with grade 0/I (Outerbridge classification modified [33]) and in 42.1% with grade II. This data underlines the possibility of an early chondropathy in those patients who are not subjected to adequate follow-up and treatment.

The comparison between the B-TP-B and the HT group through qualitative and quantitative MR analysis found no significative differences between the two groups in terms of chondropathy showing that it is not the type of ACL reconstruction that influences the onset of arthrosis. Our observation results in accordance to the systematic review of Belk et al. [14] in which no significative differences were found between the use of B-TP-B or HT for ACL reconstruction at 11 years from surgery.

In this study we evaluated the concordance between qualitative and quantitative (T2 mapping) analyses for everyone of the 27 segments by which we divided the cartilage. As suggested by other studies [21, 23], T2 mapping is a useful tool for analyse the cartilage as a whole, but it loses its diagnostic value when it comes to small segments. In particular, as Surowiec et al. [21] pointed out, the subdivision of the knee cartilage into clinically relevant and reproducible subregions such as variations in the composition and structure of the healthy cartilage, proved to be incoherent and can lead to an uneven distribution of T2 cartilage values. Furthermore, as demonstrated by Slauterbeck et al. [36] cartilage degeneration can occur throughout the entire joint, highlighting the need for subregions they take this into consideration.

Comparison of the three functional scales (KOOS, Tegner Lysholm, and IKDC) and the standard MRI assessment of the PF joint showed KOOS and Tegner Lysholm scales had significant agreement between the self-report questionnaires and the MRI results on the grading of chondropathy. As reported in previous studies [37, 38], avoidance of sports activity for fear of a new injury could explain the low pain ratings in these functional scales compared to an underlying condition of early chondropathy without clinically relevant symptoms.

Furthermore, there was no significant agreement between functional scales and the quantitative assessment of the 27 subregions through T2 mapping. This may be explained by the limitations of the T2 mapping described above, whereby T2 mapping works best when considering the cartilage as a whole rather than high-number segmentation [21, 36].

This study has some limitations. The small population surely can affect the statistical power of the study and can explain the lack of significance of T2 mapping results. Another limitation is the use of a single-vendor MRI. About the non-significative difference between the two ACL reconstruction techniques, one limit is represented by the short period underwent from the surgery, but a longer period could enlight some differences. The group of patients enlisted for the study was homogeneous for indirect factors influencing the onset of chondropathy, such as BMI, smoking habits, diabetes and hypertension. Further studies should consider a more heterogenous population. The small number of patients enlisted compromises our study.

In the end, T2 mapping is a powerful tool for quantitative analyses, but it still suffers from inter-observer bias as there is no international standard for processing the data obtained from the sequences. As suggested by Surowiec et al., to improve this issue a full automatization of the segmentation is needed [21, 28].

## Conclusions

After ACL reconstruction there is often a reduction in physical activity which could be determined by the onset of chondropathy. The advanced T2 mapping technique has proved to be a useful tool for detecting early cartilage alterations. However, the partial positivity of the functional scales suggests that psychological avoidance factors could play an important role in the reduction of sports activity.

The development of chondropathy would not appear to be linked to the type of intervention between the use of B-TP-B or HT. Furthermore, T2 mapping did not detect specific sectors within the cartilaginous layer that could be more exposed to the development of chondropathy, although a lack of a standardized protocol for the image processing.

This work represents a preliminary study, further investigation with larger sample size and different vendor machine are mandatory.
